# Extrahepatic Distal Cholangiocarcinoma vs. Pancreatic Ductal Adenocarcinoma: Histology and Molecular Profiling for Differential Diagnosis and Treatment

**DOI:** 10.3390/cancers15051454

**Published:** 2023-02-24

**Authors:** Anastasios Gkountakos, Filippo M. Martelli, Nicola Silvestris, Michele Bevere, Mario De Bellis, Laura Alaimo, Elena Sapuppo, Francesca Masetto, Aldo Mombello, Michele Simbolo, Elena Bariani, Michele Milella, Matteo Fassan, Aldo Scarpa, Claudio Luchini

**Affiliations:** 1 ARC-NET Applied Research on Cancer Center, University of Verona, 37134 Verona, Italy; 2 Department of Diagnostics and Public Health, University of Verona, 37134 Verona, Italy; 3 Medical Oncology Unit, Department of Human Pathology “G. Barresi”, University of Messina, 98125 Messina, Italy; 4 Department of Surgery, Dentistry, Gynecology, and Pediatrics, Division of General and Hepatobiliary Surgery, University of Verona, 37134 Verona, Italy; 5 Section of Medical Oncology, Department of Medicine, University of Verona, 37134 Verona, Italy; 6 Section of Pathology, Department of Medicine (DIMED), University of Padua, 35122 Padua, Italy

**Keywords:** pancreatic ductal adenocarcinoma, distal cholangiocarcinoma, diagnosis, genetic, transcriptomic, targeted therapy

## Abstract

**Simple Summary:**

Pancreatic ductal adenocarcinoma (PDAC) and distal cholangiocarcinoma (dCCA) are very aggressive neoplasms. However, effective treatments are still limited. Starting from a common embryogenesis of the tissue of origin, these two cancer types share several histomolecular features, which renders a differential diagnosis challenging. However, there are also significant differences, with a potential clinical impact. Here, we present the main similarities and differences between PDAC and dCCA, also discussing the most important implications derived from this challenging differential diagnosis.

**Abstract:**

Pancreatic ductal adenocarcinoma (PDAC) and distal cholangiocarcinoma (dCCA) are very aggressive tumors with a high mortality rate. Pancreas and distal bile ducts share a common embryonic development. Hence, PDAC and dCCA exhibit similar histological features that make a differential diagnosis during routine diagnostic practice challenging. However, there are also significant differences, with potential clinical implications. Even if PDAC and dCCA are generally associated with poor survival, patients with dCCA seem to present a better prognosis. Moreover, although precision oncology-based approaches are still limited in both entities, their most important targets are different and include alterations affecting *BRCA1/2* and related genes in PDAC, as well as *HER2* amplification in dCCA. Along this line, microsatellite instability represents a potential contact point in terms of tailored treatments, but its prevalence is very low in both tumor types. This review aims at defining the most important similarities and differences in terms of clinicopathological and molecular features between these two entities, also discussing the main theranostic implications derived from this challenging differential diagnosis.

## 1. Introduction

Pancreatic ductal adenocarcinoma (PDAC) and cholangiocarcinoma (CCA) are highly aggressive tumors with a poor prognosis [1]. PDAC is believed to originate from both ductal and acinar cells, usually via pancreatic precursor lesions [2,3,4]. CCA arises from the lining epithelium of intrahepatic and extrahepatic bile ducts and also through biliary intraepithelial precursor lesions [5]. According to the anatomical site of origin, CCA is classified into intrahepatic (iCCA) or extrahepatic (eCCA); moreover, eCCA is divided into perihilar (pCCA) and distal (dCCA) [6]. Around 60% of cases are classified as pCCA, 20–30% as dCCA and 10–20% as iCCA [7,8,9]. PDAC arising into the pancreatic head and dCCA involving the intrapancreatic tract of choledocus represent two of the most important periampullary tumors. The generalist and obsolete term of “peri-ampullary tumors” should be avoided in this setting. We adopted it for defining malignancies in the district of ampulla and adjacent tissues, but in this review, we highlight the importance of defining the exact tissue of origin. PDAC and dCCA are characterized by anatomical proximity, morphological similarity, and an overlapping immunohistochemical profile. Owing to the absence of early clinical symptoms for both cancer types, at the time of diagnosis, patients are usually presented with an advanced-stage disease and poor prognosis. Moreover, accurate clinical biomarkers are missing, and the current imaging techniques struggle to distinguish PDAC from dCCA [10,11]. The serum carbohydrate antigen 19-9 (CA 19-9) is the only FDA-approved tumor-associated biomarker used in PDAC and dCCA. However, CA 19-9 accuracy in identifying the exact disease is low, because its levels could also be affected by obstructive jaundice due to benign conditions or other pancreatobiliary diseases [12,13,14]. Therefore, a precise pre-operative diagnosis is often challenging. Artificial intelligence (AI) and one of its main branches, i.e., machine learning (ML), have entered into the field of cancer diagnostics, generating great interest. Although this field is still growing, it is widely accepted that AI and ML present considerable potential as an assistive tool in cancer detection by creating ML models based on different clinicopathological characteristics (histology, cytology, serum biomarkers) and screening tests (CT, MRI), which increases the diagnostic accuracy of both PDAC and CCA [15,16]. A pancreaticoduodenectomy (PD) with regional lymph node dissection is the standard therapeutical approach for both PDAC of the pancreatic head and dCCA [17]. Interestingly, for PDAC, neoadjuvant chemotherapy is also gaining more importance for resectable tumors [18]. Moreover, the type of chemotherapy and the response rate are different between PDAC and dCCA. Despite the aggressiveness of both PDAC and dCCA, there are also some differences in terms of survival [19]. Currently, patients with PDAC are usually treated based on FOLFIRINOX, or gemcitabine plus capecitabine regimens, whereas patients with dCCA are usually treated with cisplatin combined with gemcitabine [19,20,21,22,23]. Here, we discuss all the available information regarding the differences between PDAC and dCCA on a histological and molecular level, which define them as distinctive oncologic entities with direct implications for clinical practice.

## 2. Histological Parameters and Potential Biomarkers for the Differential Diagnosis of PDAC vs. dCCA

**Figure 1:**
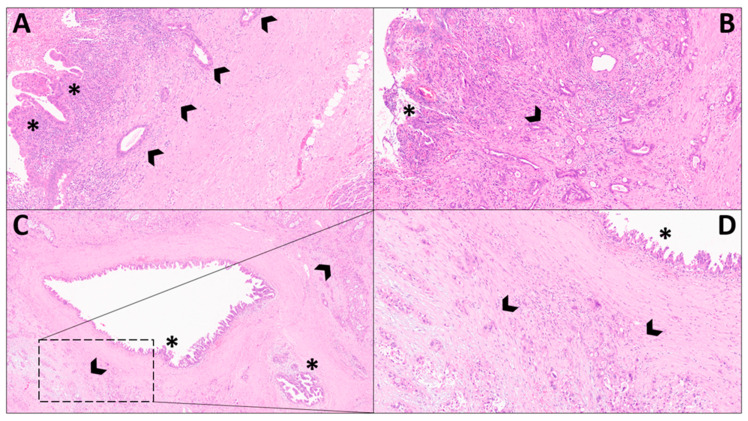
Highly illustrative figure showing the most typical histological features of distal extrahepatic cholangiocarcinoma (dCCA) and pancreatic ductal adenocarcinoma (PDAC). (**A**) This figure shows the presence of a dCCA composed of well-differentiated infiltrative glands (arrowheads), arising from a biliary intraepithelial neoplasia with high-grade dysplasia (asterisks) (Hematoxylin-eosin, original magnification: 10×). (**B**) This figure shows the presence of a dCCA growing around the choledocus and composed of moderately differentiated infiltrative glands (arrowhead); due to an extensive ulceration in the choledocus lumen (asterisk), the potential presence of biliary dysplasia cannot be evaluated. A desmoplastic stromal reaction is also evident (Hematoxylin-eosin, original magnification: 10×). (**C**,**D**) These figures show the presence of a PDAC composed of poorly differentiated infiltrative glands (arrowheads), with associated pancreatic intraepithelial neoplasia of the main and branch ducts (asterisks). A desmoplastic stromal reaction is also evident (Hematoxylin-eosin, original magnification (**C**): 4×, (**D**): 20×).

Histologically, both PDAC and dCCA are adenocarcinomas, composed of infiltrating glands with variable degrees of differentiation, ranging from good to moderate and poor differentiation [6]. Tumor cells of both neoplasms are usually atypical and show enlarged nuclei with prominent nucleoli. The stromal component is usually abundant in both PDAC and dCCA, with a desmoplastic reaction. These aspects highlight the fact that not only tumor morphology but also the associated stromal component may be indistinguishable between PDAC and dCCA. Along these lines, immunohistochemistry can also not definitively support the differential diagnosis between these two entities, with similar expressions of cytokeratin (CK), usually CK 8/18/19 positive and CK 20 negative, and of mucins (MUC), usually MUC1 positive and MUC2 negative [6]. One of the most important features for addressing a differential diagnosis is looking at the anatomical location, with PDAC centered on the pancreatic parenchyma and eCCA growing around the choledocus. Moreover, the presence of microscopic and macroscopic precursor lesions may help in identifying the actual origin of the neoplasms, with the presence of pancreatic intraepithelial neoplasia (PanIN) for PDAC [24,25], and of biliary intraepithelial neoplasia (BilIN) for dCCA [26]. The differential diagnosis between these two entities is even more challenging in the cytological/biopsy setting, in which pathologists cannot evaluate the entire lesion and its related features [27]. Figure 1 summarizes the most important histological features of both PDAC and dCCA.

Since the differential diagnosis between PDAC and dCCA is challenging, above all in the pre-operative setting, some studies have tried to address this difficult scenario testing different integrated approaches. A recent study with a retrospective design studied a cohort of patients who underwent PD for PDAC or for dCCA. The assessment of contrast-enhanced CT images taken before surgery and its integration with clinical characteristics suggested that the combination of a bile duct angle of ≤130°, a diameter of the Wirsung’s duct of ≥4.3 mm, and the absence of jaundice were significantly associated with the diagnosis of PDAC compared to dCCA [28]. Similarly, a study evaluated the bile duct axis deviation, suggesting that an angle of ≤110° could predict the presence of PDAC rather than dCCA [29]. Another study based on a cohort of 101 patients who underwent PD sought to improve the preoperative differential diagnosis by applying a diagnostic score. Patients’ data were retrospectively analyzed for obtaining a composite score based on three parameters: Wirsung’s diameter, CA 19-9, and reactive C protein. A score of 1 was assessed to each parameter in cases of Wirsung’s duct dilatation > 3 mm, CA 19-9 > 230 U/mL, and reactive C protein levels > 10 mg/Dl. These values concur to create a final score ranging from 0–3. Cases with either a 2 or 3 score value were diagnosed as PDAC, whereas those with 0–1 score were diagnosed as dCCA. This method showed a reliable specificity, but a larger number of patients are needed to confirm its potential applicability [30].

Towards the identification of alternative biomarkers, a recent study used liquid chromatography–mass spectrometry platforms for proteomic analysis, aiming at identifying candidate proteins able to differentiate PDAC from dCCA [31]. The first analysis initially found 1829 proteins expressed by both tumor types, with 15 differentially expressed proteins between PDAC and dCCA. Further analyses, including semi-quantitative comparison for validation, identified a set of five proteins that showed the best performance. Specifically, keratin 17 (KRT17), annexin 10 (ANXA10), and transmembrane protein 109 (TMEM109) were overexpressed in PDAC, whereas parathymosin (PTMS) and sodium/potassium-transporting ATPase subunit beta-1 (ATP1B1) showed a higher expression in dCCA, reaching statistical significance. In particular, KRT17 had the best performance in identifying PDAC (76.4% sensitivity, 71.6% specificity), and PTMS showed the best performance in identifying dCCA. Although these represent interesting findings, they should also be validated in biopsy samples, since such study was performed on surgically resected tumor tissues only [31].

Of note, serum levels of CA 19-9 are usually elevated in patients with pancreatobiliary malignant tumors compared with benign diseases of the same district [32,33]. Moreover, hyperbilirubinemia is a common finding in this type of tumor. Although there is no clear correlation between CA 19-9 and bilirubin levels, the neoplastic obstruction of the bile duct has been associated with increased serum levels of CA 19-9 [34]. However, it has to be acknowledged that the dynamic changes of CA 19-9 are also closely dependent on tumor stage and its biologic aggressiveness, as well as to the presence of other underlying diseases. Thus, the optimal cut offs/thresholds are still under debate. A recent study investigated the potential role of serum biomarkers in differentiating PDAC and dCCA by integrating the blood levels of CA 19-9, which is still considered the only validated biomarker for pancreatobiliary malignancies and those of bilirubin [35]. This study was based on a cohort of 265 patients, including 212 patients with cancer (178 PDAC and 34 dCCA) and 53 with benign conditions of the periampullary region. The blood levels of CA 19-9 and bilirubin were assessed for each patient before any treatment. The performance in differentiating PDAC vs. dCCA vs. benign conditions of four models (model CA 19-9/bilirubin^−1^, model CA 19-9, model bilirubin, model CA 19-9 + bilirubin) was assessed by receiver operating characteristic (ROC) curves. The model Ratio CA 19-9/(bilirubin^−1^) was the best predictor along this line [35]. This methodology, being reliable and based on routine biomarkers, could become part of the diagnostic algorithm of patients with periampullary disorders. Other recent investigations further highlighted an important role of CA 19-9 in the differential diagnosis of PDAC vs. dCCA vs. benign conditions. In particular, a study confirmed this role of CA 19-9 in combination with a signature of nine serum metabolites, including acycarnitine, ceramide, sphingomyelins, and other phospholipoproteins [36]. Along this line, two other studies have suggested that integrating the plasma levels of CA 19-9 and thrombospondin-2 could distinguish malignant periampullary tumors from benign conditions [37,38]. The following Table 1 summarizes the aforementioned factors as potential diagnostic markers.

## 3. Genetic Profiles of PDAC vs. dCCA and Targeted Therapy

The pancreatic head and the distal bile duct are characterized by a mutual development during embryogenesis, and this can explain the histomorphological similarities between these two entities [39,40,41]. Thanks to recent approaches based on massive parallel sequencing, knowledge on the genomic landscape of PDAC vs. dCCA has been drastically improved [42,43,44,45,46,47,48,49].

Regarding PDAC, around 90–92% of tumors harbor *KRAS* mutations, with G12D and G12V being the most common mutated isoforms [42,43,44]. Moreover, PDAC is also characterized by a high mutation rate of *TP53*, *CDKN2A,* and *SMAD4* [42,43,44]. Currently, only a small proportion of patients with PDAC may benefit from molecularly based therapies, and they more often belong to the subgroup of *KRAS*-wild-type tumors [50]. Indeed, although several pharmacological approaches have been attempted targeting the KRAS and MEK/ERK pathway, all these efforts failed to enhance clinical efficacy [51,52,53]. The development of KRAS G12C inhibitors, such as sotorasib and adagrasib, represent a modern revolution for patients with non-small-cell lung cancer (NSCLC) [54], but it should be acknowledged that this variant is very rare in PDAC (1%) [55]. The most promising tailored treatments for patients with PDAC based on genomic features rely on two conditions: (i) the presence of alterations affecting homologous recombination genes, including *BRCA1*, *BRCA2*, and *PALB2*, in a condition known as homologous recombination deficiency (HRD) [56,57,58]; (ii) the presence of microsatellite instability (MSI) [59,60,61]. Patients with HRD showed not only an improved response to platinum-based chemotherapy, but a subset of patients treated with PARP inhibitors achieved long-term survival [57,62]. At the same time, selected patients with PDAC harboring MSI can be treated with immunocheckpoint inhibitors, although this approach should be better refined in patients with PDAC in general [63,64,65,66,67]. It should be clarified that MSI is rare in PDAC (around 1% of cases) and is mostly associated with the medullary and mucinous/colloid PDAC variant [63]. Moreover, further studies are needed for improving knowledge on the administration of immunotherapy in patients with PDAC harboring MSI [59,61,63,64,65,66,67].

Concerning CCA, a large repertoire of different genetic alterations across the different subtypes has been described [46,47,48]. Specifically, the most frequent alterations in eCCA are represented by *KRAS*, *TP53*, *ARID1A,* and *SMAD4* mutations [48,49,68,69]. On the other hand, iCCA carries *TP53*, *IDH1*/*2,* and *ARID1A* mutations, as well as *FGFR1*-*3* alterations [70]. The new paradigms of targeted therapy in iCCA now include patients harboring *IDH1* mutations and *FGFR2* fusions [70]. Ivosidenib is an FDA-approved IDH1 inhibitor for patients with previously treated CCA carrying *IDH1* mutations [71]. Moreover, the FGFR2 inhibitor pemigatinib showed very promising results in cases harboring *FGFR2* fusions [72]. Notably, *IDH1* mutations and *FGFR2* rearrangements occur almost exclusively in iCCA and not in eCCA. Interestingly, the phase IIA study MyPathway explored the clinical activity of pertuzumab plus trastuzumab in a cohort of patients with biliary tract cancer harboring *HER2* amplification, of which 18% had eCCA. The majority of patients with eCCA treated with such a regimen showed partial response or stable disease. Given these promising results, some clinical trials are further exploring this therapeutic strategy [73]. Other potential targets for tailored approaches in eCCA are represented by MSI and *PI3KCA* alterations [48,49], but such molecular targets can be found only in a small proportion of patients.

Different transcriptomic studies have suggested further subtyping of PDAC based on the RNA expression profile [74,75,76,77]. Although each study followed different approaches, for example, in considering tumor cells only or also the stromal component, two main transcriptomic subtypes were identified. The first is the “basal-like”/”squamous” group, which exhibits a very aggressive clinical behavior. The second is termed the “classical” subtype, which is characterized by more favorable prognostic indices and seems to be more responsive to standard chemotherapy. Along this line, similar studies in eCCA have been performed, allowing for the identification of different transcriptomic subgroups also in this tumor entity [48,49]. Montal et al. identified four different transcriptomic subgroups (mesenchymal, metabolic, proliferation, immune) [48], whereas Simbolo et al. suggested that the dCCA contained two transcriptomic subgroups, one characterized by the presence of druggable alterations, and the other lacking actionable opportunities [49]. Collectively, this new molecular taxonomy combined with conventional histology might lead to the development of more efficient treatments.

## 4. Clinical Clues

Differentiating PDAC vs. dCCA has important clinical implications. A recent multi-institutional study specifically investigated this topic, including a total of 1463 patients, of which 1.239 (85%) were identified with PDAC and 224 (15%) with dCCA [78]. All patients underwent curative-intent PD. They were explored for defining potential differences between PDAC and dCCA regarding long-term survival, pathological features, and treatment parameters. Interestingly, patients with dCCA had smaller tumors with a significantly lower rate of nodal metastasis than patients with PDAC. Moreover, patients with dCCA showed a longer cancer-specific survival (CSS) compared with PDAC (39.8 months vs. 22 months, *p* < 0.001). Nodal status emerged as an important prognostic factor for both PDAC and dCCA. Importantly, adjuvant therapy was associated with improved CSS only in PDAC with nodal-positive disease. Regarding dCCA, no improvement in terms of CSS was observed, independently of nodal status [78]. Moreover, a metanalysis of 11 studies (including the aforementioned study) compared large cohorts of patients with PDAC and dCCA, exploring potential differences between these two cancer types. Indeed, patients with PDAC appear to have larger tumors with more aggressive behavior. Interestingly, dCCA had more favorable tumor pathological features and long-term prognosis than patients with PDAC. On the other hand, patients with dCCA suffer more frequently from postoperative complications and especially postoperative pancreatic fistula [79]. These findings further corroborate the idea that a correct differential diagnosis is crucial for addressing the best therapeutic strategies.

## 5. Other Peri-Ampullary Cancers

It is important to recognize that, in addition to PDAC and dCCA, there are other types of peri-ampullary tumors, which challenge the differential diagnosis and treatment of malignancies arising in this district. They are: (i) ampulla of Vater carcinoma (AVC) and (ii) duodenal carcinoma (DC) [6].

AVC represents up to 20% of peri-ampullary tumors [6,80,81]. The Ampulla of Vater is an anatomical region with unique histological aspects, since it represents a crossroads of three different epithelia: intestinal, ductal pancreatic, and biliary. Thus, neoplasms arising in this area can show morphological complexity and heterogeneity. Histologically, the most common subtypes of AVC are intestinal, pancreatobiliary, and mixed [6]. Of them, the most challenging differential diagnosis with PDAC and dCCA is represented by the pancreatobiliary subtype. This tumor is characterized by the presence of complex tubular glands composed of atypical cells and is associated with a prominent desmoplastic stroma [6]. At immunohistochemistry, cells stain positively for MUC1, MUC5AC, and CK 7 [6,81]. The importance in correctly recognizing this tumor entity derives from its enrichment in potentially actionable molecular targets. Indeed, MSI is present in up to 20% of AVC, although its prevalence is higher in the intestinal subtype [60,82]. Moreover, *ERBB2* amplification has been detected in up to 23% of cases [83,84,85]. In a recent study, it was observed in 13% of AVC, regardless of histology, and it was mutually exclusive with downstream mutations in *KRAS*/*NRAS*/*BRAF*, which are responsible for resistance to therapies targeting *ERBB2* [85]. Based on these observations, immediately after a diagnosis of AVC, the assessment of MSI and of *ERBB2* status should be performed as reflex test. Of note, MSI is quite common in AVC [60,86]; it is more often observed in the intestinal subtype, but also the pancreatobiliary subtype can harbor such a molecular alteration [86].

DC is a rare tumor arising in the extra-ampullary portion of duodenum [6]. Similar to AVC, this cancer type can also be subdivided into different histotypes, which are gastric, intestinal, and pancreatobiliary [87]. Different from AVC, the latter is reported with a lower frequency, however [6,87]. Still, it represents a challenging differential diagnosis with AVC, PDAC, and dCCA in cases of periampullary involvement. In the case of DC, too, a correct differential diagnosis is important and should be based on specific histomolecular correlations. Of note, regarding genetic alterations, DC is enriched in MSI, which should be assessed by a reflex test immediately after the diagnosis of DC.

Interestingly, peri-ampullary-region adenocarcinoma with an indeterminable origin (PRAIO) might represent another entity of peri-ampullary tumors. The PRAIO extends along both the bile duct and the main pancreatic duct and is characterized by unique morphological features, different from those of PDAC, dCCA, or other peri-ampullary tumors. However, studies with a larger number of PRAIO cases should be performed towards the establishment of PRAIO as a distinct entity [88].

## 6. Liquid Biopsy and Its Diagnostic and Prognostic Role in PDAC and CCA

Given the challenges that characterize the sequential collection of PDAC and CCA tissue biopsies, the concept of liquid biopsy as a minimally invasive and simple tool represents a promising alternative option for diagnostic, prognostic, and predictive purposes. Across several cancer types, liquid biopsy has paved the way for improving clinical management, including early diagnosis, appropriate treatment selection, and monitoring of the disease [89]. Following the constant advances in the era of precision medicine, the molecular profiling of tumors is becoming more important, starting from its prognostic value [90]. Today, next-generation sequencing (NGS) and high-throughput sequencing platforms are widely used for mutation analysis of liquid biopsy-related material, such as circulating tumor cells and tumor DNA (ctDNA) [91]. Currently, despite the considerable number of promising findings, the adoption of liquid biopsy has not yet entered into clinical practice for pancreatobiliary tumors, but there are already very promising results in terms of its prognostic and predictive role.

For instance, a study in which the MSI was assessed by Guardant360 (a liquid biopsy-related laboratory test) in the plasma of nine patients with PDAC and treated with ICI (pembrolizumab or ipilimumab plus nivolumab) reported an overall response rate of 77% (7/9) [92]. Interestingly, tracking the changes within tumor masses using liquid biopsy might provide insights into tumor heterogeneity, evolution, and response to treatment. Indeed, recent evidence has shown that longitudinal monitoring with liquid biopsy of surgically resected patients with PDAC could reveal the presence of *KRAS* ctDNA, and it correlated strictly with a poorer prognosis. Moreover, no detection of *KRAS* ctDNA patients was significantly correlated with a better response to first-line chemotherapy [93]. Along this line, other studies have confirmed the role of *KRAS* ctDNA detection and its association with different clinicopathological characteristics, thus suggesting *KRAS* ctDNA as a circulating biomarker for prognosis and response to therapy [94,95,96].

Regarding CCA, a large study performed an NGS-based analysis of 2068 cell-free DNA (cfDNA) samples from 1671 patients with CCA. Of them, 84% harbored at least one mutation, and 44% presented at least one actionable alteration [97]. Next, the mutational concordance regarding three different actionable alterations between paired samples of tissue and cfDNA of 194 patients was explored. *IDH1* mutations were detected in the tissue of 47 patients. Of those patients, 87% (41/47) harbored *IDH1* mutations also in cfDNA. *BRAF* V600E was detected in only four patients. Interestingly, there was a 100% concordance between tissue and cfDNA. Regarding *FGFR2* fusions, such an alteration was detected in tissue specimens of 67 patients. However, this result was confirmed in only 18% (12/67) of cfDNA samples [97], highlighting the current difficulties in liquid biopsy-based methodologies in detecting structural rearrangements rather than single nucleotide variations. Similarly, a comparison of the tissue-ctDNA mutational profiles of 23 patients with CCA reported that the general concordance was 74%. Specifically, the concordance rate was 92% for iCCA, but only 55% for patients with eCCA [98]. Interestingly, a recent study also showed that bile fluid is rich in tumor-derived material, indicating that this might act as complementary or surrogate source of plasma. Indeed, bile samples from 42 patients with CCA (36/42 with eCCA) were acquired, and matched tissue specimens and plasma samples were collected from 20 (17/20 eCCA) and 16 (15/16 eCCA) patients, respectively. The extracted ctDNA was analyzed for *KRAS* mutations using droplet digital PCR. Mutations of *KRAS* were identified in 20/42 bile samples, with an 80% overall agreement between matched bile ctDNA and tissue samples and a 42.9% overall concordance between plasma and tissue samples [99]. Along those same lines, a study compared the mutation profiling of bile and plasma samples from 28 patients with CCA (8/28 with dCCA) analyzed with NGS-targeted sequencing for 520-related genes. The bile-derived cfDNA proved to be superior to plasma cfDNA in detecting somatic mutations. Specifically, somatic mutations were detected in 71.4% (20/28) of bile samples, a larger proportion compared to 53.6% (15/28) of plasma samples [100].

With the considerations regarding liquid biopsies and the intrinsic difficulties in obtaining tissue samples in the clinical setting of pancreatobiliary malignancies, this new tool might represent a promising perspective for improving the management of patients affected by such neoplasms, and above all, for detecting potential targets for tailored treatments. Indeed, for the differential diagnosis between PDAC and dCCA, it may suffer due to the genetic similarities of the two entities, as above in the case of KRAS assessment. Of note, in the era of precision medicine, cfDNA could serve as a surrogate for tissue, overcoming the limitations of traditional biopsy [101], thus mimicking the already established paradigm of assessing *EGFR* status in NSCLC in the pancreatobiliary district [102].

## 7. Conclusions

The differential diagnosis between PDAC and dCCA is an important task and represents the first step for addressing the best therapeutic choice for a patient with a tumor mass growing into the pancreatic head. As previously shown, although some improvements in imaging-based techniques should be acknowledged, PDAC and dCCA are not easily distinguishable preoperatively. Interestingly, the application of AI has increasingly been gaining ground in cancer radiology and could offer new solutions with innovative AI-based technologies for analyzing MRI and CT scans [103].

Due to their common developmental origin, PDAC and dCCA share several important histopathological characteristics. Moreover, PDAC and dCCA are usually refractory or only partially responsive to chemotherapy, and patients have a poor median survival. Therefore, early diagnosis remains a crucial point for both tumor types. Of note, as also highlighted by the information presented here, there is a strong rationale indicating the importance of distinguishing PDAC vs. dCCA. The advent of multi-omics technologies has drastically improved our knowledge of these tumors. Although some important similarities between PDAC and dCCA should also be acknowledged at the molecular level, the genomic and transcriptomic profiles of PDAC and dCCA show significant differences, with potential implications on tailored treatments. Notably, at the molecular level, PDAC and dCCA are both characterized by the presence of very few druggable alterations, which include MSI and HRD for PDAC and MSI, *HER2* amplification, and *PI3KCA* alterations for dCCA.

Considering the site of origin/anatomical position of PDAC and dCCA, the collection of tumor samples of sufficient quality/quantity is not always guaranteed. Therefore, alternative sources of DNA should be also explored, including liquid biopsy, which represents a promising future perspective along this line. Importantly, PDAC vs. dCCA distinction and the taxonomy of the different types of CCA should be clearly defined in every study. As a result, the most important characteristics will be immediately available and correctly attributed to the different tumor types, accelerating the correct recognition of PDAC and dCCA as distinct entities and ultimately improving therapeutic approaches.

## Figures and Tables

**Table 1:** List of biomarkers with potential PDAC vs. dCCA diagnostic role.

Potential Biomarker	Methodology	Role	Refs.
BDA ≤ 130°, PDD ≥ 4.3 mm, absence of jaundice/ BDA ≤ 110°	Radiographical-based BD axis deviation	PDAC > dCCA	[28,29]
Wirsung duct dilatation > 3 mm, CA 19-9 > 230 U/mL, CRP > 10 mg/DI	Preoperative diagnostic score	dCCA (0 or 1) vs. PDAC (2 or 3)	[30]
KRT17+/ANXA10+/PTMS− KRT17−/ANXA10−/PTMS+	LC-MS/proteomics	PDAC > dCCA dCCA > PDAC	[31]
Model CA 19-9/bilirubin^−1^	Blood levels of CA 19-9 and bilirubin	PDAC vs. dCCA	[35]
9-metabolites signature + CA 19-9	Serum metabolic profile	PDAC vs. dCCA	[36]

Abbreviations: PDAC, pancreatic ductal adenocarcinoma; dCCA, distal cholangiocarcinoma; BDA, bile duct angle; PDD, pancreatic duct diameter; BD, bile duct; CA 19-9, carbohydrate antigen 19-9; CRP, C-reactive protein; KRT17, keratin 17; ANXA10, annexin 10; PTMS, parathymosin; LC-MS, liquid chromatography–mass spectrometry.
